# Sexual assault survivors’ perspectives on clinical follow-up in the Eden District, South Africa: A qualitative study

**DOI:** 10.4102/phcfm.v10i1.1631

**Published:** 2018-05-30

**Authors:** Gail Holton, Kate Joyner, Robert Mash

**Affiliations:** 1 Eden District Department of Health, George, South Africa; 2 Department of Nursing and Midwifery, Stellenbosch University, South Africa; 3 Division of Family Medicine and Primary Care, Stellenbosch University, South Africa

## Abstract

**Background:**

Although effective follow-up of sexual assault survivors is linked to optimal recovery, attendance at follow-up consultations is poor. It is therefore essential that health care providers maximise the benefit of follow-up care for every sexual assault survivor.

**Aim:**

This study explored the personal experiences of sexual assault survivors to better understand the enablers of, and barriers to, attendance at follow-up consultations.

**Methods:**

This phenomenological qualitative study was conducted at the three hospitals which manage most sexual assault survivors within the Eden District. Using purposive sampling, 10 participants were selected. Consenting participants shared their experiences during semi-structured interviews with the researcher.

**Results:**

Authoritative, client-held documentation was a powerful enabler to accessing follow-up care. Individualised, patient-centred care further enhanced participants’ access to, and utilisation of, health care services. The failure of health care providers to integrate follow-up care for sexual assault survivors into established chronic care services was a missed opportunity in the continuum of care. Negative perceptions, based on others’ or personal prior experience of police, judicial and health care systems, were further barriers to follow-up care.

**Conclusion:**

This study highlights the need of survivors of sexual assault for integrated, patient-centred care, encompassing principles of good communication. Committed actions of all stakeholders are necessary to tackle negative perceptions that create barriers to follow-up care. A simple practical strategy, the provision of a scheduled appointment on official stationery, is easy to effect at facility level. As a powerful enabler to follow-up care, this should be implemented as a priority intervention.

## Introduction

The World Health Organization (WHO) estimates that one in three women worldwide experiences physical or sexual assault during their lifetime.^1^ Survivors of sexual assault are at increased risk of developing serious short- and long-term physical, mental, sexual and reproductive health problems.^1^ Support and care from others; however, assists sexual assault survivors to initiate the process of recovery and build adaptive coping behaviours and resilience.^2^

In South Africa, as few as one in 25 incidents of sexual assault is reported to the police.^3^ Many survivors only access health care after reporting the violation. It is therefore essential that effective, quality, health care services are available to those accessing the facilities.

A comprehensive report by Röhrs^4^ highlights the lack of comprehensive adherence counselling, fragmented management, the persistence of negative attitudes, long waiting times and the lack of written information for survivors as barriers to care. It was noted that survivors expressed a need for care by competent and empathetic staff.^4^ A positive response to an increased and individualised follow-up care plan emphasised the importance of psychosocial support post sexual assault.^5^

Various methods of improving follow-up care have been explored. In the Democratic Republic of Congo, community-based services were introduced in response to perceived barriers to accessing health services.^6^ Implementing mobile clinic services thereby taking health care to the community resulted in a significant improvement in attendance at the first follow-up consultation.

The concept of a specialised ‘one stop’ treatment centre has been adopted at some South African facilities known as Thuthuzela Care Centres.^7^ These projects are collaborations between various stakeholders, a primary link being the National Prosecuting Authority. Although considered a best practice, there is no reference to improved follow-up care of survivors at Thuthuzela Care Centres.^7^

Guidelines recommend sexual assault survivors be followed up one week, six weeks and three months after the initial consultation.^8,9^ Follow-up services include monitoring and review of post-exposure prophylaxis, human immunodeficiency virus (HIV) and pregnancy screening, contraception choices, counselling and referral to specialised services.

Providing quality follow-up care plays a role in limiting the negative consequences of sexual violation.^10^ Available data indicate that attendance at follow-up consultations is low, about 35.5%.^11^ A similar pattern in Eden District suggests challenges to ongoing care within the current services.

This study explored the personal experiences of sexual assault survivors from three sites within the Eden District to better understand the enablers of, and barriers to, attendance at follow-up consultations. The study was intended to provide useful information and evidence to inform future care strategies and change management aimed at improving follow-up care.

## Methods

### Study design

This phenomenological qualitative study utilised semi-structured interviews to explore sexual assault survivors’ experiences.

### Setting

Knysna District Hospital, Mossel Bay District Hospital and George Regional Hospital were selected as study sites as they manage the majority of the sexual assault cases within the Eden District. This provided the best opportunity for participation while still reflecting the diversity of community and hospital services. Forensic services for sexual assault survivors were provided exclusively via these government facilities. Although accessible to everyone, the service was predominantly accessed by the uninsured population. George Hospital houses the only Thuthuzela Care Centre within the district. During office hours, psychosocial support was provided by Thuthuzela staff. After hours, counselling was covered by community partners. Health care services were provided by the hospital’s emergency centre doctors within the Thuthuzela examination room. All follow-up consultations were provided by the hospital’s occupational health and safety professional nurse. In Mossel Bay Hospital, survivors were triaged via the emergency centre. Clinical examinations were conducted by the doctor on call within a specifically designated room, and community partners were integrated into psychosocial support. During office hours, the hospital social worker counselled all survivors and coordinated follow-up care. Knysna Hospital provided the only service offered by forensic nurses. The hospital is equipped with a modern, designated rape crisis facility. Forensic nurses are supported by the emergency centre doctor on call if necessary. A senior family physician provided on call services in rotation with two forensic nurses. The forensic nurses facilitated all follow-up consultations.

### Selection of participants

The study population comprised all sexual assault survivors with mobile numbers entered into the designated hospitals’ registers 3–6 months prior to the interview. During this period, participants would have had the opportunity to attend at least three post-assault follow-up sessions. The 6-month cut-off aimed to limit challenges with memory recall. Survivors were eligible for study participation irrespective of their attendance at follow-up consultations. Exclusion criteria for the study were profoundly or severely intellectually disabled or psychotic patients who were unable to give a coherent interview. Eligible survivors were phoned consecutively as they appeared in the register and interviewed if they agreed. The intent was to interview four participants at each study site, incorporating approximately 20% of the monthly caseload. Data saturation was achieved when no new content was expressed during participant interviews, namely when emerging themes became repetitive, as reflected in the researcher’s field notes.

### Data collection

Field work was conducted between April and June 2016. Following informed consent or assent, the researcher, assisted by a multilingual (English, Afrikaans and isiXhosa) interpreter, conducted the interviews using an interview guide. Key topics included survivors’ experiences of the initial consultation, follow-up instructions, and enablers and barriers to follow-up care. The researcher’s role as the District Women’s Health Coordinator is non-clinical, thus facilitating objectivity. The interpreter was an experienced counsellor recognised for her sensitivity and competence. A peer review team was formed to support the study processes and its trustworthiness. Each team member contributed a specialised skill set.

### Data analysis

Applying the framework method, interview transcripts were thematically analysed using Atlas.ti software.^12^ Participant numbers replaced names to maintain confidentiality and anonymity. Analysis systematically adhered to the following steps: familiarisation with the data, creation of a thematic index, systematic coding of all transcripts, charting of data with the same codes and finally interpretation. The researcher compiled the thematic index and analysed the findings. K.J., as supervisor, confirmed the analysis, thereby providing consensus prior to completion of the report.

### Ethical considerations

Ethical approval was provided by the Health Research Ethics Committee of Stellenbosch University (S15/10/225). The Research Division of Strategy and Health Support, Western Cape Department of Health granted access to the study sites (WC_2016RP56_169). Written informed consent, including permission to record the interviews, was obtained prior to the commencement thereof. Parents of minor participants (< 18 years) gave consent with the minor providing informed written assent.

## Results

The study sample comprised 10 female survivors. Three of these were girls aged 9, 10 and 15 years, respectively. To minimise the risk of secondary trauma, the grandmother and father, as primary caregivers of the younger girls, were interviewed by the researcher. The 15-year-old girl was interviewed with her mother present at her request. This participant was part of a nuclear family and both parents were supportive. The 9- and 10-year-old children had relocated from mother-led family units to their grandmother and biological father, respectively. They attended formal schooling and lived in formal dwellings. Three young women aged 18–22 were also interviewed. The 18-year-old attended high school as a boarder and lived with her older sister when not at school. The other two were employed full time and lived with their parents in formal, low-cost houses. The remaining four participants were women aged 32–42, two of whom had left their employment because of post-traumatic stress. One of the participants had relocated to a neighbouring town, and another participant relocated to a different area within the same town. They both rented rooms in low-cost housing and had no formal income. The 42-year-old woman resided in a night shelter at the time of the interview. She depended on a disability grant for income. Four participants received care at Knysna Hospital, four at George Hospital and two at Mossel Bay Hospital.

### Health care system issues

The importance of an individually tailored care plan encouraged attendance at scheduled consultations despite financial and transport constraints. Participants preferred empathic continuity of care with a familiar, trusted health care provider at follow-up. Some expressed dissatisfaction at not seeing the same provider because of staff leave or rotation. This feeling was compounded if the alternative provider was male. Although participants preferred to be cared for by female providers, this was superseded by their understanding that a professionally qualified and competent provider was essential, regardless of gender:

‘I want to go to the sister again because the doctor didn’t explain nicely to me. I thought I was going to see that sister again, and then I saw it was a man. I was embarrassed.’ (Participant 1, female, 15 years old)

**TABLE 1:** Summary of themes.

Themes	Sub themes	
	Enablers	Barriers
Health care systems	Patient-centred care	Primary health care reception Lack of confidence in the health service Waiting times
Police systems	Empowering documentation	Distrust Inability to provide safety Discouraging of reporting
Judicial system	None identified	Slow and unresponsive system Perceived leniency towards perpetrators Despondency with the system’s capacity to effect resolution
Health care provider factors	Positive experience at initial consultation Formal appointment made	No follow-up appointment made Unmet health care expectations
Community response	Catalyst for accessing health care	Judgemental response towards survivor Concern about reprisal as a result of vigilante type community support
Client factors	Passive compliance	Internal conflict: post-traumatic stress symptoms Relocation as a means of coping and ensuring ongoing safety Work or school obligations Financial constraints

All 10 participants told of a positive initial encounter with the sexual assault health care service. Survivors voiced their relief and appreciation of a caring, thorough initial consultation. Participants preferred minimal, but reassuring, conversation with the provider during the initial consultation:

‘Hmm, he [*doctor*] worked alright with me, gently, and also didn’t hurt me. Every now and then he spoke a few words. … It was enough because at that time I didn’t feel like talking, not a lot anyway.’ (Participant 4, female, 32 years old)

Many participants experienced challenges when interacting with the primary care reception staff. As gatekeepers to primary care services, the reception staff played a critical role in creating barriers between the client and service:

‘She says that her treatment wasn’t good at reception. She was told that Sister [*name withheld*] was not there – but not in a nice way.’ (Participant 3, female, 19 years old)

As the primary care facilities are located within the community, staff are often well known or even related to these survivors. Lack of privacy, potential loss of confidentiality and the proximity of the primary care clinic to the area where the sexual assault took place were identified as barriers to accessing follow-up care:

‘It was difficult … at reception. If I talk … they will know the reason why I am coming to the clinic. Do you understand?’ (Participant 2, female, 32 years old)

### Health care provider issues

The provision of a scheduled, documented appointment emerged as a critical enabler to follow-up care. All five participants who had been given formal follow-up appointments attended follow-up services. Some survivors were not provided with any information regarding the need for follow-up, and others were only given verbal instructions to attend the local clinic. Those who had not been given a scheduled follow-up appointment did not attend:

‘No, I asked him [*doctor*] must I come again and he said: No you must not come again, I must just take her to the clinic for an HIV test. And then I didn’t go.’ (Participant 1, female, 15 years old)

Accessing health care facilities without recognised documentation prompted unwarranted scrutiny from front-line staff, in some instances the security personnel at the entrance gate:

‘And you also can’t go without a letter, because he [*doctor*] can’t just say you must go to the clinic or you must come back. He must write a date and must give you a letter to be able to go to the clinic.’ (Participant 4, female, 32 years old )

For participants employed or attending school, set appointments enabled them to negotiate and plan time off to attend follow-up appointments. Noting all three follow-up dates on the appointment card assisted planning. The provision of an official appointment was further strengthened by a clear, detailed explanation of the proposed health care plan by the health care provider.

Follow-up instructions provided for HIV-positive survivors were vague. HIV-positive survivors were expected to access follow-up care via their existing HIV care systems. Participant 10, a regular chronic health care client, indicated the reluctance of her usual provider to engage with the topic of sexual assault even though it was documented in the medical record:

‘They did give him the information about what happened to me, but he has still never asked me, “how do you feel about this business, how are you coping? How can I help you get back to your life, to help build you up and try and forget about this case?”’ (Participant 10, female, 42 years old)

### Police service issues

The case document, issued to the survivor by the police, was identified as an enabler. The document provided encouragement and a sense of purpose, enabling some survivors to overcome their vulnerability and take proactive measures to assert themselves.

In general, participants expressed their satisfaction with the specialised police service of the Family Violence, Child Protection and Sexual Offences (FCS) unit. Barriers identified related to police personnel not from this specialised unit. Distrust for the police was a common theme. Personal experience and anecdotal accounts of community dealings with the police fuelled distrust. This was compounded by the opinion that the police were unable to ensure the survivor’s safety. Participants reported alleged perpetrators not adhering to bail conditions. Some participants relocated to avoid proximity to the alleged perpetrator, inadvertently creating a barrier to accessing follow-up health care.

Participants reported secondary victimisation by police. The validity of their claim of sexual assault was questioned, thereby discouraging reporting and minimising the severity of the violation:

‘Because they [*police*] asked me, the day [*name withheld*] was raping me, did I call anyone? I said no. So that’s why the guy [*police officer*] said he doesn’t think they’re going to take me seriously because I didn’t cry.’ (Participant 7, female, 32 years old)

### Community responses

The community’s reaction to the participants’ disclosure of sexual assault significantly affected the survivors’ motivation and self-esteem. In many instances, the community acted as a catalyst by assisting the survivor’s access to initial health care. Participants also told how community members provided clothes, called the police and stayed with them until the police arrived.

‘I was running in the street. My clothes were still in that house. So this guy just came. I don’t know where he was coming from. He just gave me a jacket so that I could cover myself.’ (Participant 6, female, 22 years old)

Conversely, there were negative accounts of overzealous community support, resulting in a vigilante-style response. Some participants indicated that this was problematic, as the community action meant they not only feared for their own safety, but for those apparently responding on their behalf. To downplay or diffuse the situation, they withdrew charges and in some instances relocated. Ironically, instead of assisting the survivor, this community action and apparent solidarity with survivors compromised their ability to access and utilise health care.

### Client factors

A common theme was an attitude of unquestioning compliance. Survivors became passive beneficiaries of care through acceptance of the health care provider’s authority. Participant 3 shared the following statement through the interpreter:

‘She went there because the doctor told her to go to the clinic, so that is why she went there.’ (Participant 3, female, 19 years old)

Participants seemed to focus more on overcoming the physical challenges and practical issues in their personal circumstances rather than their psychological well-being. Physical health was dealt with, but more abstract concepts of emotional, psychological and spiritual health were neglected. Insomnia, difficulty concentrating, flashbacks, self-blame and self-isolation were commonly discussed. Some spoke of turning to drugs and alcohol to escape from the painful reminders of the sexual violation. Participants experienced severe challenges with concentration and information retention. This was evident during the initial consultation where providers had given verbal instructions regarding taking of medication and follow-up care. Unable to concentrate, the survivors could not remember these verbal instructions. The absence of written instructions created a barrier to follow-up care:

‘Yes. When someone is talking to you, your mind is not there. You hear she is talking, but you don’t know what she’s saying.’ (Participant 7, female, 32 years old)

Some participants felt follow-up sessions were superfluous. These participants felt they were coping and did not see the point of attending follow-up sessions:

‘I don’t need any follow-ups. I don’t need anything. For my personal life I need this [*prosecution of perpetrator*].’ (Participant 6, female, 22 years old)

Participants identified work and school obligations as barriers to follow-up care. Financial constraints, specifically transport costs, were noted. However, the participants who identified these challenges overcame them and attended scheduled follow-up consultations.

### Judicial system issues

No enablers of follow-up regarding judicial systems were suggested. Challenges identified included leniency and an inability to ensure that bail conditions for alleged perpetrators were enforced. Participants expressed despondency with the judicial system. Without the assurance of a valid claim, survivors were discouraged and did not prioritise follow-up health care:

‘When she saw my folder, it was written in red that she should tell me that the case has been postponed because I could not identify the men. I asked her even though I can’t identify their faces did you not find anything [*evidence*] on my clothes. Is there no indication of who it could be? Then she said to me, lady, sorry there is nothing I can do about that.’ (Participant 2, female, 32 years old)

## Discussion

All participants who had been provided with follow-up appointments on official stationery adhered to their scheduled appointments. Although strikingly obvious, this enabler is perhaps the most critical finding of the study. As a barrier, this highlights an operational gap easily remedied without high-level intervention. Apart from the expectations linked to scheduled appointments, it conveys a tangible commitment to continuity of care, enabling the survivor to effectively access the facility. Further, the provision of a police case document had a positive impact on the sexual assault survivor. Validation of the survivor’s claim of sexual assault provided encouragement and a sense of purpose, enabling attentiveness to her health care needs.

A patient-centred approach was a central enabler of this study and included relational continuity of care, being attended to by the same trusted health care provider, and an individualised treatment plan. The Patients’ Rights Charter^13^ indicates that continuity of health care should be guaranteed. Knowing and trusting the health care provider encouraged participants to attend scheduled follow-up consultations.^14,15^ These findings highlight the need to include the survivor in the planning of their treatment programmes. Unfortunately, appropriate patient-centred skills to facilitate this mutual decision-making process appear to be lacking. This may be because of inadequate training in generalist skills that capacitate health care providers’ implementation of holistic care.^16^ Missed opportunities to combine patients’ needs for follow-up of sexual assault with other routine health care also suggest a challenge in the understanding of comprehensive, integrated care.

Reassuringly, all participants indicated their satisfaction with the initial consultation, providing a potential platform for continued utilisation of health care services. Positive patient care experiences are associated with higher levels of adherence to treatment processes.^15^

The findings revealed that survivors who were HIV positive at the time of the assault were not prioritised for follow-up care. Faced with highly stressful workloads,^16^ health care providers may not have provided HIV-positive survivors with a follow-up appointment because they assumed that the survivor was already included in a chronic care system. Alternatively, it suggests health care staff may only have focused on the processes linked to post-exposure prophylaxis and did not perceive the importance of comprehensive follow-up encompassing physical, psychological, emotional and spiritual health.

The reluctance of regular health care providers to engage with their clients holistically may represent a lack of confidence or prioritisation or a notion that mental health care requires specialised intervention. A mental health section has been incorporated into the Western Cape standard guidelines entitled, ‘*Practical Approach to Care Kit* (PACK): Primary Care Guideline for Adults 2016–2017’.^17^ Mental health screening in primary health care has been included as a reporting data indicator for 2017–2018. The integration of mental health into primary care should encourage health care providers to implement a holistic approach. However, the gulf between policy and the comprehensive implementation thereof remains a significant challenge.

Negative experiences during prior interactions with front-line facility staff, concerns about confidentiality and facility proximity to the scene of the assault reveal that current primary care systems pose barriers to follow-up consultations. The mere possibility of an unpleasant experience with the health care system created a barrier to the utilisation of follow-up services.

Although the service is ostensibly free, the ‘hidden’ costs were significant for survivors with low or no income. Public transport services in the Knysna and Mossel Bay sub-districts are absent, making survivors dependent on the sometimes unscrupulous charges and timetables of the taxi industry. However, despite these challenges, participants who identified transport costs as a barrier demonstrated their resilience by overcoming them and attending follow-up.

Similar findings emerged within the police and judicial systems. Distrust in the services negatively impacted their utilisation of follow-up services. The need for recognition, justification and confirmation that the survivor’s claim of sexual assault was valid was paramount. This represents a valuable finding of the study.

Findings indicate a sinister pattern of police discouraging reporting and convincing the survivor that there was insufficient evidence to proceed with the case. This under-reporting may be attributed to the pressure on the police service to show an annual decrease in crime.^4^ In addition, participants found that police were unable to ensure their personal safety through their inability or reluctance to enforce bail conditions for alleged perpetrators. The doubt cast on the survivor’s claim of sexual violation negatively impacted their uptake of follow-up care.

There may also be benefits of training community members to respond to survivors in a kind and supportive manner.^18^ Confirmation by the community that the survivor was justified in her allegation of sexual assault provided much needed psychological support. Having the crime validated by external people legitimised the survivors’ need for care.

Negative social reactions to sexual assault disclosure can be associated with symptoms of post-traumatic stress disorder among survivors.^19^ Judgemental, disbelieving views reinforce and thus promote stigma and blame, entrenching the connotation that the survivor is somehow responsible for the sexual violation. This extends to the intended community support through vigilante-style retribution. In some instances, this action created a barrier to follow-up care rather than the intended support. Altering attitudes and interactions is complex, but not insuperable as demonstrated by the Raising Voices study in Uganda.^20^ Maximum benefit requires an all-inclusive call to action by civil society, backed up by legislation that is enforceable and health care that serves clients’ needs.

### Strengths and limitations

The poor response to calls and accuracy of cell phone details in the sexual assault register limited the sample population. Many mobile phone numbers provided were incorrect, went straight to voicemail or belonged to someone other than the survivor. On one occasion, the case manager from the Thuthuzela Centre phoned 11 contact numbers and only reached one survivor. Nevertheless, no new themes were emerging by the eighth, ninth and tenth interview. Consequently, sufficient saturation of data was achieved.

During recruitment, it became apparent that the majority of male adult sexual assault survivors presenting to the George and Mossel Bay hospitals were inmates of correctional service facilities. As inmates, these survivors had no control over their attendance at follow-up services. Correctional services managed post-assault follow-up consultations in-house. As the inmates had no choice, and their access to follow-up services was entirely dependent on the correctional services processes, the experiences of these adult males were missed.

### Recommendations

The implication of this study encourages health care providers to return to the basic principles of holistic care. Enabling access to, and utilisation of, follow-up care post sexual assault is the collective responsibility of every service-based programme, community member and individual.

The provision of scheduled appointments on an official document is of primary importance. This should be supplemented with a standardised referral system incorporating written information for both the survivor and health care provider’s reference. Clinical governance methods that prioritise the ability of health care providers to offer comprehensive, holistic, patient-centred follow-up care post sexual assault should be developed. Innovative methods to register accurate contact details for survivors would be beneficial. Action research could be used to develop training programmes and implementation strategies to improve consultation competencies in primary care. It could also explore what sexual assault survivors actually want, need and benefit from, while simultaneously changing clinical practice. In terms of inmates, this study exposes a topic worthy of further investigation.

## Conclusion

The enablers of access to follow-up care for sexual assault survivors identified by participants revealed practical and achievable interventions. A collaborative, integrated and patient-centred approach to follow-up care is vitally important. Client-held official appointment cards and the provision of police case numbers were identified as powerful enablers. Identified barriers included unhelpful behaviours and attitudes from community members, police, judicial and health care workers. Although these barriers are more difficult to remedy, they are important in the process of improving care to survivors of sexual assault.
